# Fabrication of multifunctional wool textile using the synthesis of silver-modified N-doped ZnO/TiO_2_ photocatalysts

**DOI:** 10.1016/j.heliyon.2024.e36522

**Published:** 2024-08-17

**Authors:** Amir Behzadnia, Majid Montazer, Mahnaz Mahmoudi Rad, Madineh Rastgoo

**Affiliations:** a Department of Textile Engineering, Science and Research Branch, Islamic Azad University, Tehran, Iran; b Department of Textile Engineering, Functional Fibrous Structures & Environmental Enhancement (FFSEE), Amirkabir University, PO Box 15875-4413, Tehran, Iran; c Skin Research Centre, Shahid Beheshti University of Medical Sciences, Tehran, Iran

**Keywords:** N-Ag/TiO_2_/ZnO, Wool, Sonosynthesis, Self-cleaning, Photoactivity, Antimicrobial

## Abstract

Photocatalysts and noble metals have attracted considerable attention for their potential in addressing global environmental pollution through photochemical processes. At low temperatures, multifunctional self-cleanable wool fabric was developed through green photo-sonosynthesis of N–Ag/TiO2/ZnO. A narrower bandgap of the hybrid photocatalyst, the surface plasmonic resonance effect of silver nanostructures, and nitrogen doping resulted in synergistically enhanced self-cleaning activity. The self-cleaning activity was evaluated by monitoring the discoloration of methylene blue stains on the wool fabric exposed to natural sunlight, using CIELAB color space and ΔE measurements. The ΔE value of the N–Ag/TiO_2_/ZnO-sonicated wool was superior, showing a value of 45.9 compared to 15.7 for the control and 28.7 for the sample coated by the stirrer. Furthermore, the nanocomposite construction improved tensile strength, enhanced fabric hydrophilicity, and reduced the yellowness index. Additionally, the synthesis of TiO_2_ and silver particles on ZnO particles increased surface resistance to acid, reducing ZnO acid solubility. The reflectance of the non-treated wool ranged from 5 to 20 % within 200–380 nm, while the reflectance of the Ag/TiO_2_/ZnO-sonicated sample remained constant at 4 %, exhibiting protection against UV rays. AATCC test revealed 100 % bacteria reduction against *E. coli* and *S. aureus* and 99 % against *C. albicans* fungus for N–Ag/TiO_2_/ZnO-sonicated sample. Moreover, cell culture assays demonstrated a viability of over 70 %, indicating non-cytotoxicity.

## Highlights

• Photo sonosynthesized of N–Ag/TiO_2_/ZnO photocatalysts on wool through in situ synthesis method.
• Relative acid resistance nanocomposite by reducing the acid solubility of nano ZnO.
• Obtaining the highest self-cleaning and antimicrobial activities on wool.
• Lower alkali solubility with higher tensile strength.

## Graphical abstract

**Figure:**
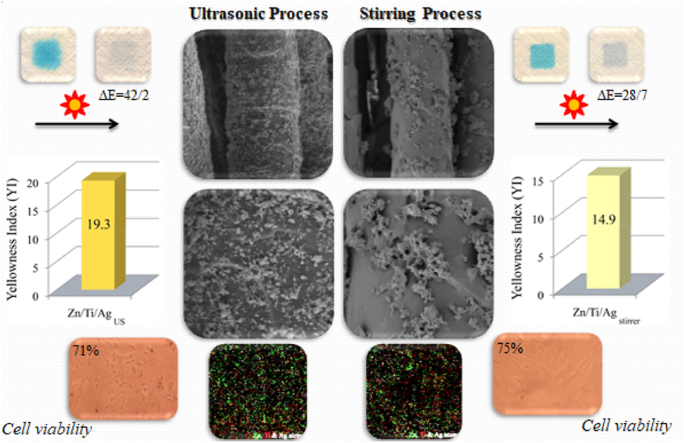

## 1 Introduction

Multifunctional and smart textiles have been extensively studied through the application of photocatalysts, particularly to achieve antibacterial and self-cleaning properties [1,2]. Using textile fabrics as a substrate for photocatalysts is also advantageous due to their flexibility and wettability, which help prevent secondary pollution from difficulties in separation after use and the agglomeration of nanoparticles [[3], [4], [5]]. Previous studies have demonstrated the application of various photocatalytic materials, such as metal oxides and noble metals, on textiles [[6], [7], [8]]. For instance, cotton textiles coated with ZnO nanoparticles have shown self-cleaning, UV protection, and antimicrobial activities [9]. Similarly, self-cleanable plasma-treated cotton fabric was produced using ultrasonic synthesis of TiO_2_ nanoparticles with Aloe vera extract [10,11]. To further enhance the photocatalytic activity of TiO_2_, Saleem et al. developed a Cu_2_O/TiO_2_ nanocomposite on textiles using a one-pot sonochemical process [12].

The unique properties of wool, including its complex morphology with multilayered cuticle and cortex, make it a promising textile template for developing photocatalytic materials [13,14]. The keratin proteins in wool contribute to its chemical resistance, strength, moisture permeability, and porosity. Its diverse functional groups facilitate various binding mechanisms, making it suitable for nanoparticle functionalization and enabling the production of fabrics with antimicrobial, self-cleaning, and UV-protective properties [15,16]. Additionally, as a protein fiber, wool contains active groups such as amide, disulfide, and carboxyl, which can reduce metal salts to form nanoparticles [17].

Previous studies have utilized ZnO and TiO_2_ nanoparticles as photocatalysts on wool fabrics [[13], [14], [15],^18^]. However, the focus on UV-induced photocatalyst textiles limits practical applications under sunlight irradiation. Additionally, complex deposition techniques such as sputtering are costly, and heat treatment in hydrothermal methods leads to grain growth and particle agglomeration [15,19]. Sonication was introduced as a green chemistry method to synthesize nanomaterials at low temperatures and improve the kinetic of chemical reactions [[20], [21], [22], [23], [24]]. Montazer et al. applied binary photocatalysts on wool through a sonochemical approach and achieved textiles with considerable self-cleaning and antibacterial activities [19,25,26]. Previous research has focused on single-component photocatalysts or binary composites, which have shown limited multifunctional capabilities.

This study aims to overcome this limitation by depositing an innovative N–Ag/TiO_2_/ZnO composite on wool fabric. Incorporating N–Ag/TiO_2_/ZnO on wool leads to the composites with abundant photoactive sites and superior self-cleaning activity under sunlight compared to their counterparts. The matched pair of energy bands in the TiO_2_–ZnO hybrid makes it possible to eliminate photoelectron-hole recombination and synergistic enhance light absorption [27,28]. Similar ionic radii and energy states of nitrogen and oxygen suggest that nitrogen doping further improves the photocatalytic activity of the ZnO and TiO_2_, making the composite effective under visible light [[29], [30], [31], [32], [33]]. Silver exhibits a broad light absorption spectrum due to Plasmon excitation efficiency [14]. Different Fermi levels of Ag and metal oxide semiconductors, as well as the formation of the Schottky barrier between them, reduce the rate of recombination between charge carriers and increase light harvesting [32,34,35].

Here, we demonstrated that N–Ag/TiO_2_/ZnO composite can be photo-sonosynthesized at low temperatures and in a short period, achieving a multifunctional wool composite with superior activities. This study combines ultraviolet and ultrasound irradiation, along with a sol-gel process, to hydrolyze zinc acetate, silver nitrate, and titanium isopropoxide, and deposit them on wool fabric. A variety of intrinsic wool fabric properties were enhanced, including tensile strength, alkali solubility, and hydrophilicity. Coated wool fabrics reported to have self-cleaning properties were not just caused by the presence of photocatalysts, but also by a hydrophobic surface that was created during the deposition of metal oxides. However, this study found that treated fabrics with photocatalysts had a significantly higher self-cleaning activity and hydrophilicity than raw fabric. Notably, self-cleaning analysis was conducted under sunlight, a practical and cost-effective approach rarely explored in the literature. Furthermore, UV protection and antimicrobial activity were demonstrated in the composite-coated textile.

## 2 Experimental

### 2.1 Preparation of photocatalyst hybrid coated fabric

**Fig. 1:**
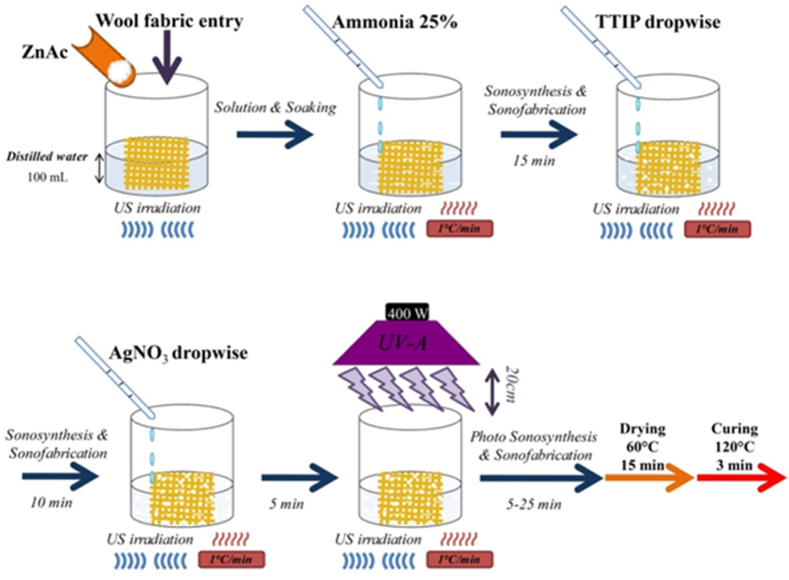
Schematic of preparation procedure for in situ photo sonosynthesis of N-doped Ag/TiO_2_/ZnO nanocomposites onto wool fabric.

The N–TiO_2_/ZnO were sonosynthesized onto the wool fabric (Iran Merinos), following our previous report, which involved the hydrolysis of zinc acetate (ZnAc, Merck) and titanium tetra isopropoxide (TTIP, Merck) under ultrasound bath at pH = 9–10 for 25 min [29]. The process was followed by introducing silver nitrate (AgNO_3_, Merck) into the sonication bath. The solution was exposed to ultrasonic and ultraviolet irradiation to synthesize silver nanoparticles on N–TiO_2_/ZnO for 15 min. At the same pH, the temperature was elevated to 75–80 °C. Finally, the sonoloaded fabric underwent a 15-min drying process at 60 °C followed by a 3-min curing step at 120 °C (Fig. 1). Following this treatment, the functionalized wool fabric was washed three times with water to be sure loose or unbound nanoparticles were removed from the fabric. The experiment conditions were also carried out to produce Zn, Ti, Ag/Zn, Ag/Ti, and Ti/Zn to study the efficiency of the products (Table 1). Ag/Ti/Zn-stirred sample was synthesized without sonication to observe how ultrasonic waves affect Ag/Ti/Zn. Also, the pH and ultrasonic treatment were studied on the NH_3_ sample to determine their effects on wool fabric quality (Table 1).

**Table 1:** Various wool fabric samples preparation.

Sample	TTIP (V/V %)	ZnAc (W/V %)	AgNO_3_ (W/V × 10^−3^ %)	UV-A irradiation (min)
Blank	–	–	–	–
Ammonia	–	–	–	–
Ti	1.5	–	–	–
Zn	–	3.5	–	–
Ti/Zn	1.5	3.5	–	–
Ag/Ti	1.5	–	6.00	15
Ag/Zn	–	3.5	2.75	15
Ag/Ti/Zn_stirred_	1.5	3.5	2.75	15

In the central composite design (CCD), thirteen experimental runs were conducted for doped fabric with ZnAc, TTIP, and AgNO_3_. Table 2, demonstrates how AgNO_3_ and UV-A exposure time affect the properties of these fabrics including, alkaline solubility (Y1), char residual (Y2, percent), water spread time (Y3, s), acid solubility (Y4, percent), self-cleaning activity and yellowness of treated fabric (Y5 and Y6, after seven days of exposing to sunlight).

**Table 2:** Experimental conditions for wool fabrics preparation and various responses based on CCD.

Run^a^	AgNO_3_ (W/V × 10^−3^ %)	UV-A irradiation (min)	Char residual (%)	Self-cleaning (ΔE)	Yellowness index		Acid solubility (%)	Alkali solubility (%)	Water spread (s)
					Before sunlight	After sunlight			
Ag/Ti/Zn_1_	2.75	15	17.0	41.2	28.8	19.3	20.3	11.6	1
Ag/Ti/Zn_2_	4.35	22	17.9	45.9	34.0	24.4	6.4	10.5	13
Ag/Ti/Zn_3_	2.75	15	17.1	41.3	28.9	19.4	20.3	11.7	1
Ag/Ti/Zn_4_	5.00	15	17.9	45.3	35.9	26.0	8.6	10.1	23
Ag/Ti/Zn_5_	1.15	8	16.0	39.3	28.4	18.0	4.3	12.3	1
Ag/Ti/Zn_6_	2.75	25	17.4	44.0	30.9	22.4	17.1	11.4	1
Ag/Ti/Zn_7_	2.75	5	16.8	41.2	28.9	19.6	18.2	12.0	1
Ag/Ti/Zn_8_	1.15	22	16.4	40.9	30.2	21.4	48.1	11.9	2
Ag/Ti/Zn_9_	2.75	15	17.0	41.2	28.9	19.4	20.3	11.6	1
Ag/Ti/Zn_10_	4.35	8	17.5	43.5	33.0	23.9	7.7	11.0	15
Ag/Ti/Zn_11_	2.75	15	17.1	41.2	28.8	19.3	20.4	11.7	1
Ag/Ti/Zn_12_	0.50	15	15.8	39.0	29.9	19.7	66.7	12.0	5
Ag/Ti/Zn_13_	2.75	15	17.1	41.3	28.8	19.3	20.3	11.7	1

a All runs contain 1.5 (V/V %) TTIP and 3.5 (W/V %) ZnAc.

### 2.2 Characterization of coated fabric

The crystalline states of coated compounds on the fabrics were studied using x-ray diffraction (XRD; STOE STAI MP) with Cu source (40 KV, 40 mA). Field-emission scanning electron microscope (FE-SEM; MIRA3-TESCAN) was used to examine the distribution and formation of N–Ag/TiO_2_/ZnO nanocomposite. X-ray analysis (EDX; Rontec) of sono and stirred samples was performed to demonstrate the immobilized samples with N–Ag/TiO_2_/ZnO nanocomposite. The elemental composition of employing photocatalysts on fabric was studied using X-ray photoelectron spectroscopy (XPS; X-Ray 8025-BesTec) using Al Ka radiation (hm = 1486.6 eV).

The MB solution was prepared with a concentration of 100 mg/L in distilled water and 0.2 mL of the solution was applied to the fabric samples (2 cm × 2 cm) to study their self-cleaning activity after 42 h under natural sunlight in Tehran (average temperature of 34 °C and average monthly relative humidity of 32 %). The discoloration of the MB samples was assessed according to Eq (1) [36].

(1) ΔE = [(ΔL*)^2^ + (Δa*)^2^ + (Δb*)^2^]^1/2^

Where, ΔE represents the total color difference, and ΔL*, Δa*, and Δb* represent the change in the color index of fabrics before and after exposure to sunlight.

To assess the ultraviolet protection provided by the synthesized nanoparticles on wool fabric, both the treated and untreated fabric samples underwent UV exposure for the measurement of their reflectance spectra within the 200–800 nm range.

As part of the analysis of the hydrophilicity of the treated and untreated samples, the spreading time of water drops on the fabric was measured according to the AATCC 79–2000 procedure. The residual char percentage after burning was calculated by weighing the samples before and after 3 h of heating at 600 °C [25]. Wool fiber's alkaline stability was assessed using an IWTO-4-66(D) test [18]. Since ZnO is highly acid-soluble, coated samples with TiO_2_ and Ag have been studied to determine if they impact its acid solubility by immersing samples in acetic acid and measuring weight changes [19]. The treated fabrics were tested for tensile strength and yellowness using ASTM D1294 and ASTM D1925, respectively.

The AATCC 100–2004 test method was used to determine the bactericidal and fungicidal activities of the treated fabrics against pathogenic microorganisms such as *E. coli*, *S. aureus*, and *C. albicans*. Those samples were placed on agar plates containing a microbial suspension, and the reduction of colonies on various fabrics was measured. The results were presented as a percentage, derived from the count of microorganism colonies recovered from the wool samples after inoculation and incubation. In addition, the MTT assay was used to determine the toxicity of samples [26].

## 3 Results

### 3.1 Formation and characterization of N–Ag/TiO_2_/ZnO photocatalysts on wool fibers

Due to the aqueous medium sonication, the radicals (H^•^ and ^•^OH) generated in the media led to the subsequent sonolysis of water within the cavitation bubble when transient collapse eventually caused recombination of radicals produced H_2_ and H_2_O_2_ species based on Eqs. (2), (3), (4) [37], led to the sonochemical hydrolysis of ZnAc and TTIP in an alkaline medium.

(2) H_2_O → H• + •OH

(3) H• + •H → H_2_

(4) HO• + •OH → H_2_O_2_

Also, the alkali solution containing silver nitrate produced silver oxide via OH- ions reacting with Ag + ions. As a result, Ag nanoparticles were formed by ultrasonic and UV irradiation through possible mechanisms based on Eqs. (5), (6), (7), (8), (9), (10), (11) [[38], [39], [40], [41]]:

(5) $${\text{Ag}}^{+}+H•\;\overset{US}{\to }\;\text{Ag}{}^{\circ}+H^{+}$$

(6) $${\text{AgNO}}_{3\;}+{\text{NH}}_{3}+H_{2}O\;\overset{US,UV}{\to }\;\text{AgOH}+{\text{NH}}_{4}{\text{NO}}_{3}$$

(7) $$2\text{AgOH}\;\overset{US,UV}{\to }\;{\text{Ag}}_{2}O+H_{2}O$$

(8) $${\text{Ag}}_{2}O\;\overset{US,UV}{\to }\;2{\text{Ag}}^{+}+O_{2}^{‐}$$

(9) $${\text{Ag}}^{+}+H_{2}O\;\overset{UV}{\to }\;\text{Ag}{}^{\circ}+H^{+}+\text{OH}•$$

(10) $$\text{OH}•+–{\text{CH}}_{2}\;\left(\text{OH}\right)\;\overset{UV}{\to }\;–\;\text{CH}•\;\left(\text{OH}\right)+H_{2}O$$

(11) $${\text{Ag}}^{+}+–\;\text{CH}•\;\left(\text{OH}\right)\;\overset{UV}{\to }\;\text{Ag}{}^{\circ}+–\text{CH}=O+H^{+}$$

The formation of N–Ag/TiO_2_/ZnO composite involves the generation of various reactive species and nanoparticles through sonochemical and photochemical reactions. Wool fibers contain hydroxyl, carboxyl, and amino groups, and TiO_2_ tends to bind specifically to the hydroxyl and carboxyl groups [42]. The functional groups on wool facilitate the binding of the photocatalysts and improve light absorption. The H• and •OH radicals, and the nanoparticles formed during the synthesis process can create hydrogen bonds or electrostatic interactions with the functional groups on the wool surface. The synthesis of silver, zinc oxide, and titanium dioxide nanoparticles on wool fibers promotes cross-linking within the wool fibers enhancing the mechanical strength and the available surface area for photocatalytic reactions.

The successful synthesis of N–Ag/TiO_2_/ZnO was verified by XRD analysis and XPS spectrum. Despite the low concentration of nitrogen, it was not detected in the XRD data.

**Fig. 2:**
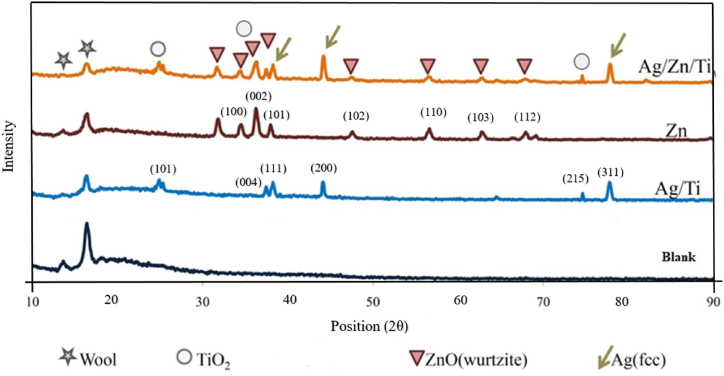
XRD patterns of different samples.

**Fig. 3:**
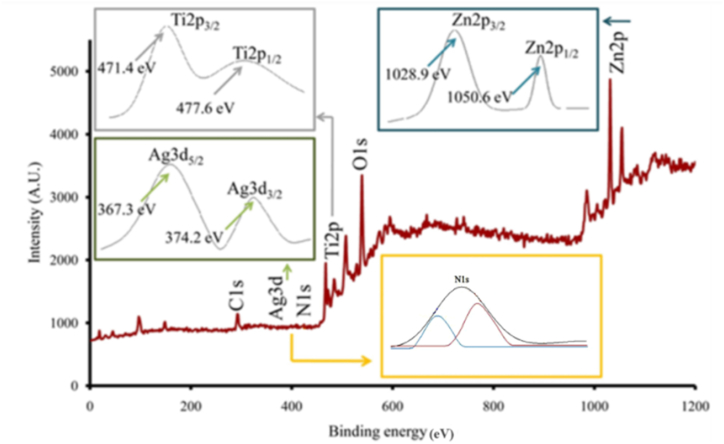
XPS spectra of in-situ N–Ag/TiO_2_/ZnO photo sonosynthesized nanopowder and corresponding high-resolution XPS spectra of Ag3d, N1s, Ti2p and Zn2p.

Fig. 3 is shown the XRD patterns of the both raw and coated samples, revealing peaks at 2θ values of 25.2° and 75.1°, corresponding to anatase [32], a peak at 36.3° for ZnO [25], and peaks at 38.3°, 44.5°, and 77.4° for silver [43]. These findings suggest that the composite consists of TiO_2_, ZnO, and Ag nanoparticles. According to Fig. 2, peak intensities of 13.7 and 16.5° correlated to wool functional groups which are lower for sonotreated than raw wool. X-rays can be prevented by the distribution of sonoproduced particles on wool. The wool fabric consists of both crystalline and amorphous structures, with its distinct crystalline peaks in the 2θ of 10–20° in XRD diffraction [44]. The untreated wool shows an amorphous broad peak starting at 20°, whereas the composite-treated wool displays distinct crystalline peaks, indicating the deposition of nanoparticles on the wool fibers. The binding of TiO_2_ to the carboxyl and hydroxyl groups on wool and the incorporation of ZnO and Ag nanoparticles can be realized from the observed XRD diffraction pattern.

To investigate whether nitrogen/silver elements were successfully doped on TiO_2_/ZnO nanocomposite particles, N–Ag/TiO_2_/ZnO samples were analyzed by XPS (Fig. 3). Therefore, elements of Ag, N, Ti, and Zn were proved to exist in co-doped nanocomposites by four peaks at binding energies of 370.4, 400.5, 474.3, 1038.7 eV related to Ag3d, N1s, Ti2p, and Zn2p, respectively [32].

The high-resolution Ag_3_d spectrum illustrates two peaks related to Ag_3_d_5/2_ (367.3 eV) and Ag3d_3/2_ (374.2 eV) bonds that prove the metallic (Ag°) state of silver elements [45].

**Fig. 4:**
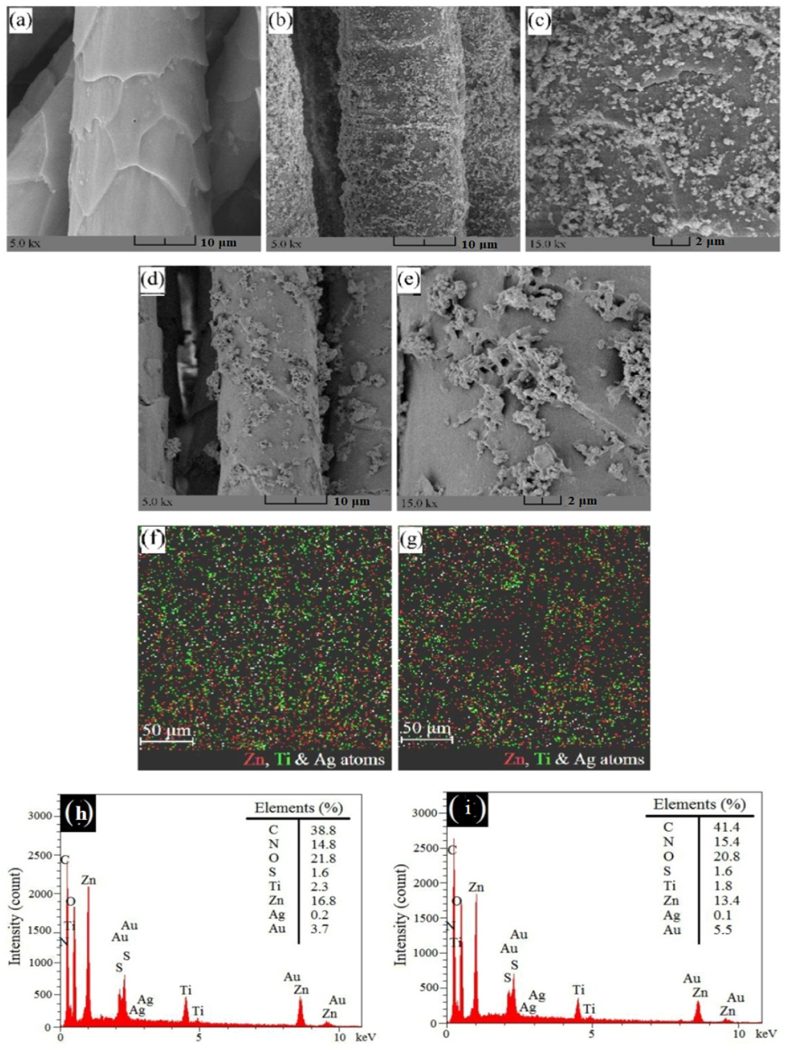
FE-SEM images of various wool samples (a) raw, (b–c) Ag/Ti/Zn1, and (d–e) Ag/Ti/Zn-stirred. X-ray mapping images of samples indicating the presence of Zn, Ti and Ag atoms (f) Ag/Ti/Zn1, and (g) Ag/Ti/Znstirred; EDS spectra of (h) Ag/Ti/Zn1, and (i) Ag/Ti/Zn-stirred.

There are two peak positions at 471.4 and 477.6 eV corresponding to Ti2p_3/2_ and Ti2p_1/2_, respectively [46]. A binding energy peak for Zn2p could be found at 1028.9 and 1050.6 eV, which are linked to Zn2p_3/2_ and Zn2p_1/2_, respectively (Fig. 4). The 21.7 eV split between Zn2p_3/2_ and Zn2p_1/2_ proves Zn^2+^ state in Wurtzite ZnO state [36].

This suggests the availability of more reactive sites for photocatalysis [32]. In examining nitrogen doping within the composite, in the N1s high-resolution spectrum, the peak at ∼400 eV suggests the presence of molecular nitrogen on the surface, indicating N interacts with Ag, ZnO, and TiO_2_, providing more reactive sites [47,48].

A lower binding energy (∼399 eV) suggests incorporating Ti with N via the TiN valence bond (K). The peak at ∼400.5 eV indicates successful N binding with Ag, while ∼402 eV is attributed to Zn3N2, demonstrating its strong association with Zn [32].

Fig. 4a indicates the raw wool surface with overlapping scales. Based on FE-SEM images of ultrasonic-treated samples (Fig. 4b–c), a uniform N–Ag/TiO_2_/ZnO nanocomposite can be observed on their surface. On the other hand, Fig. 4d.

-e shows the clustered-like of crystalline N–Ag/TiO_2_/ZnO nanocomposites on the surface of fibers that formed during in situ photosynthesis and the post-heating process.

EDX analysis of sono and stirred treated samples, namely Ag/Ti/Zn1 and Ag/Ti/Zn-stirred was studied (Fig. 4f and g). The ratio of elements presented on the raw wool fibers and the treated fabric is illustrated in Fig. 4h and i. A sonotreated sample has a more uniform distribution of zinc, titanium, and silver elements than a stirred sample.

### 3.2 Self-cleaning and UV protection activities

A color variation analysis was carried out on stained pieces with MB by measuring the color values (L*a*b*) prior to and following and after seven days of exposure to sunlight, Table 1.

**Fig. 5:**
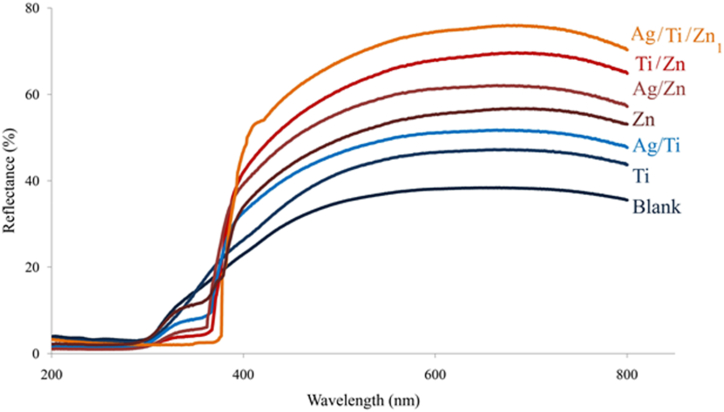
UV reflectance spectra of various samples.

The UV reflectance spectrums of blank, Ti, Ag/Ti, Zn, Ag/Zn, Ti/Zn, and Ag/Ti/Zn1 were illustrated at 200–800 nm (Fig. 5). The results indicate that sonotreated samples with N–Ag/TiO_2_/ZnO nanocomposite have a higher UV absorption than samples treated with other compounds. In addition to absorbing UV rays, the nanocomposite of N–Ag/TiO_2_/ZnO prevents ultraviolet degradation and photo-yellowing of fabric.

### 3.3 Mechanical and physical analysis

Table 2 presents the averages of tensile strength and elongation with CV%, indicating increased tensile strength for TTIP and ZnAc treated wool by 13 and 18 %, respectively, compared with raw wool fabric.

Further, Ag nanoparticles onto N–TiO_2_ and N–ZnO led to a higher tensile strength of about 4 % and 3 % for Ag/Ti and Ag/Zn, respectively. N–TiO_2_/ZnO nanocomposites also enhanced tensile strength from 503 for NH_3_ to 647 N for Ti/Zn. Adding Ag onto N–TiO_2_/ZnO nanocomposites resulted in a 28 % increase in tensile strength, achieving a superior tensile strength of 663 N, which indicates higher strength compared to previous reports on nanocomposite wool fabric. For example, TiO_2_/Ag/SiO_2_ coated wool increased tensile strength by 16 %, while selenium nanoparticles treated wool increased tensile strength by 11.27 % [17].

The yellowness index (YI) of all treated samples are presented in Table 2, Table 3. The best whiteness belongs to the sample with the least YI. N–Ag/TiO_2_/ZnO structure is produced as a cream-brown color solution. Also, more AgNO_3_ in the sonobath under ultraviolet with prolonged time leads to a darker solution. However, sono and stirred treated samples with N–Ag/TiO_2_/ZnO composite under sunlight irradiation indicate lower YI due to plasmonic resonance and narrow bandgap leading to more visible light absorbance generating more e^−^/h^+^ pairs on the surface of fabrics. More nanoparticles are fabricated on the fabric surface with more precursors in the sono-bath, resulting in a lower yellowness index. This confirms the formation of the finer particles that are possibly inserted into the fiber structure.

**Table 3:** Different responses of various wool samples.

Sample	Acid solubility (%)	Char residue (%)	Self-cleaning (ΔE)	Yellowness index		Alkali solubility (%)	Water spread (s)
				Before sunlight	After sunlight		
Blank	–	0.2	15.7	39.7	49.9	30.7	>120
Ammonia	–	0.1	15.6	38.6	51.0	31.6	>120
Ti	–	1.6	30.3	34.0	21.6	18.3	110
Ag/Ti	–	1.7	35.4	51.4	40.1	17.9	75
Zn	100	12.2	34.3	29.9	19.8	13.1	45
Ag/Zn	48.2	12.9	37.2	40.0	32.5	12.6	20
Ti/Zn	50.5	15.6	42.2	27.6	11.2	12.2	1
Ag/Ti/Zn_stirrer_	55.2	10.0	28.7	27.2	14.9	19.7	>120
Ag/Ti/Zn_2_	6.4	17.9	45.9	34.0	24.4	10.5	13

The residual ashes were established to calculate the various sample char residues (Table 2, Table 3). In this experiment, the fabric was burned as a sub-layer to assess the stability of loaded materials at high temperatures. Raw wool char residue (0.2 %) improved to 16.6 % for Ti/Zn and 17.0 % for Ag/Ti/Zn1. The results show the positive effect of synthesized particles on wool fabric to increase char residue.

The alkali solubility of the treated samples indicated lower alkali solubility after loading Ag nanoparticles on N–TiO_2_/ZnO (Table 2, Table 3). Alkali hydrolysis of wool involves breaking the cysteine group of wool chains, resulting in the disulfide bond separation. More AgNO_3_ as a precursor in impregnation sonobath produced lower alkali solubility. Furthermore, the alkali solubility of stirred Ag/Ti/Zn exhibited a higher value than Ag/Ti/Zn1 because ultrasound stimulates the diffusion of nanoparticles on fibers uniformly.

The acid-resistant test is established to determine the decomposition of sonoprepared ZnO in acid media. A lower acid solubility was observed after loading both N–TiO_2_ and Ag nanoparticles onto N–ZnO (Table 2, Table 3). Acid hydrolysis leads to topochemical degradation at the surface of ZnO, resulting in particle separation. However, N–TiO_2_ and Ag particles incorporated into N–ZnO result in self-supported nanocomposites. The acid solubility of N–ZnO (100 %) sharply decreased to 50.5, 48.2, and 20.3 % for Ti/Zn, Ag/Zn, and Ag/Ti/Zn1, respectively. As a result of ultrasound irradiation, by adding nanoparticles of N–TiO_2_ and Ag onto the surface of N–ZnO, the acid solubility of Ag/Ti/Zn1 is lower than Ag/Ti/Zn-stirred.

Wool samples showed greater hydrophilicity with ultrasound irradiation. Acoustically treated wool fabrics absorb water droplets more quickly than stirred samples. Due to the ultrasonic treatment, smaller nanoparticles became situated on the surface of the wool fabric (Table 2, Table 3). Further, the presence of Ag in Ag/Ti and Ag/Zn compounds shortens the time taken for water absorption compared to Ti and Zn alone.

### 3.4 Antimicrobial and cytotoxicity activities

As shown in Table 4, a range of samples including Ti, Ag/Ti, Zn, Ag/Zn, Ti/Zn, Ag/Ti/Zn1, and raw wool was tested for antibacterial and antifungal activities in the suspension method. It was demonstrated that synthesis with TTIP and ZnAc alone produced low antimicrobial activity compared with treated fabric with silver in the photo sonobath (Ag/Ti and Ag/Zn). Thus, by introducing Ag nanoparticles to N–TiO_2_/ZnO nanocomposites, greater antimicrobial activity was achieved, as similarly demonstrated in earlier reports [18,47]. The silver intrinsic bactericidal and fungicidal properties and the ability to trap photo-excited electrons may play a part because of the inhibition of electron-hole recombination. The *order of antimicrobial properties is:*

**Table 4:** Antibacterial/antifungal, cell viability, and mechanical properties of different wool samples.

Sample	Antibacterial/antifungal (%)			Cell-viability (%)	Mechanical properties (±CV%)	
	*E.coli*	*S.aureus*	*C.albicans*		Tensile strength (N)	Elongation (mm)
Blank	0	0	0	100	516.4 ± 4.8	37.9 ± 9.8
Ammonia	0	0	0	100	502.9 ± 7.1	29.5 ± 9.0
Ti	55	41	35	89	577.2 ± 7.1	29.1 ± 6.8
Ag/Ti	74	53	42	81	600.3 ± 4.2	23.5 ± 9.3
Zn	91	86	75	87	608.5 ± 7.2	22.4 ± 7.9
Ag/Zn	94	90	83	76	625.4 ± 4.1	21.2 ± 8.2
Ti/Zn	95	91	68	77	647.2 ± 4.6	18.3 ± 4.6
Ag/Ti/Zn_1_	100	100	99	71	663.1 ± 3.2	16.9 ± 7.7

*E. coli (Gram-) > S*. aureus (Gram+) > *C. albicans* (fungi) [48].

TTIP, ZnAc, AgNO_3_, and ammonia-treated fabric were examined for their cytotoxicity on human dermal fibroblasts. This was achieved by adding an extraction medium to the cells and measuring their cell viability through MTT analysis. As a control, cells without samples had a viability of 100 %, and after 24 h incubation, cells over raw wool and sonotreated samples had a viability of 70–100 % (Table 4). As per ISO 10993-5, the extract of a treated sample with an average cell viability of over 70 % is considered biocompatible [19]. Photos of cells were used to assess how toxic agent affects the shape and metabolism of the cells [49]. Most sonotreated fabrics with TTIP, TTIP/AgNO_3_, ZnAc, ZnAc/AgNO_3_, ZnAc/TTIP, and ZnAc/TTIP/AgNO_3_ kept the initial cone shape after incubation, although small morphological changes were observed in some cells. Moreover, sonotreated samples with nanocomposites demonstrated lower cell viability when compared to nanoparticles alone, and no significant cytotoxicity was observed on human skin. The ammonia sample also maintained 100 % cell viability.

## 4 Discussion

### 4.1 Sonochemical and photochemical synthesis of the hybrid photocatalysts on wool

In the current study, the two-synthesis approach was applied to develop multifunctional wool fabric by the formation of N–Ag/TiO_2_/ZnO nanocomposite through ultrasound-assisted synthesis and UV photo-reduction of nanoparticles [[50], [51]]. The effects of cavitation bubbles generated through N–Ag/TiO_2_/ZnO reactions in sonobath are as follows [19]:

(1) Water molecules dissociate into OH radicals during the sonoluminescence mechanism; generated radicals produce H_2_O_2_ that oxidizes hydroxide to oxide [19,21].

**Fig. 6:**
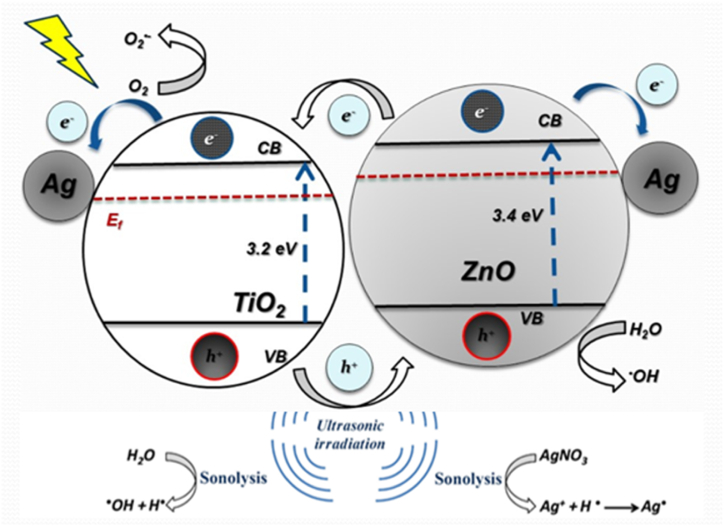
A schematic of sonosynthesis of robust N–Ag/TiO_2_/ZnO nanocomposites through sonoluminace generation under ultrasonic irradiation and formation of electrons/holes on the interface of particles.

Firstly, the bubble nucleation can promote cavitation, leading to the formation of hot spots in the solution, at which high temperatures have reached that cause the generation of OH• from H_2_O. Secondly, sonoluminescence, caused by ultrasound waves, includes UV light resulting from cavitation. The excited TiO_2_ and ZnO particles generate OH• (Fig. 6) [19,26,52].

(2) During intercollisions of bubbles, metal oxide particles formed from metal alkoxide, acetate, and nitrate hydrolysis flow at high speeds, causing "microcalcination", resulting in photo sonosynthesis of the N–Ag/TiO_2_/ZnO photocatalyst.
(3) At the interface of transient bubbles, during their collapse, the liquid shell near the bubble can be heated and undergo microcalcination leading to N–Ag/TiO_2_/ZnO composite formation [19].

**Fig. 7:**
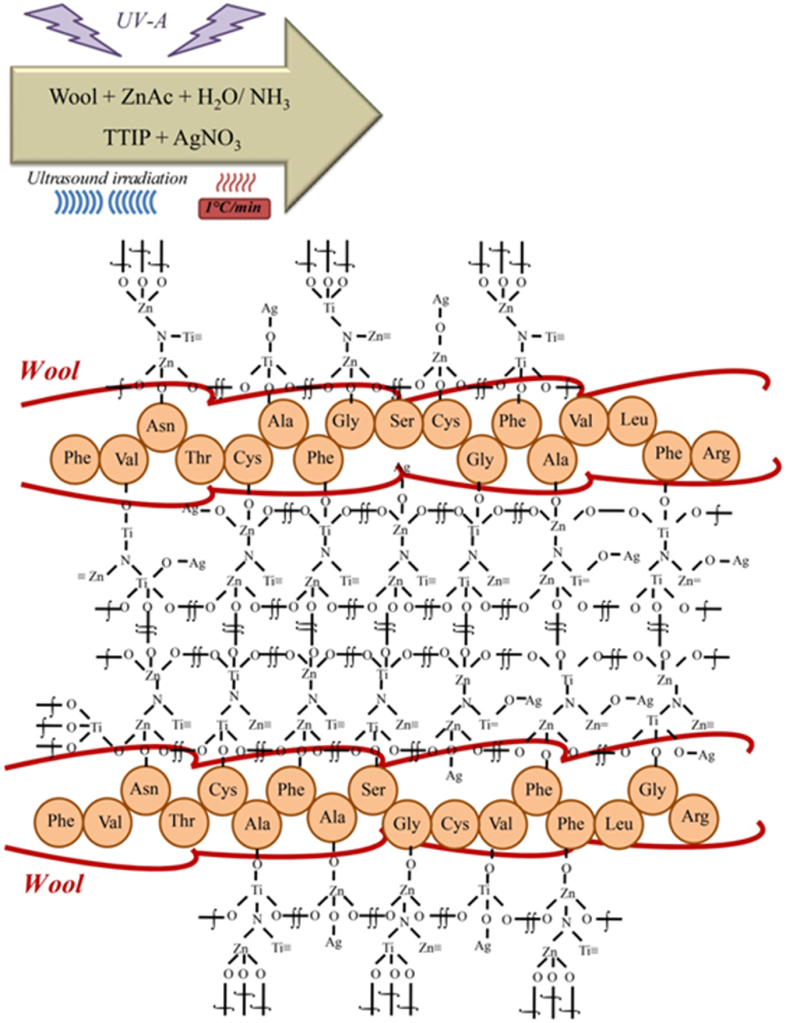
The schematic mechanism of synthesis of N–Ag/TiO_2_/ZnO nanocomposites on wool fiber.

As a result of UV irradiation during the synthesis of nanoparticles, free radicals are formed, which then cause the photoreduction of Ag, resulting in silver nanoparticles. UV irradiation causes Ag + ions reduction, generates smaller nanoparticles, and improves nanoparticle attachment on wool fabric by forming reactive species [53]. As an added benefit, functional groups of wool (e.g., amino, phenol, hydroxyl, cysteine, thiol) also served effectively as reactive sites, resulting in tensile strength improvement when the N–Ag/TiO_2_/ZnO composite and the functional groups of wool react (Fig. 7).

### 4.2 Self-cleaning activity under sunlight

The self-cleaning performance of samples under sunlight irradiation is shown in Table 2, Table 3 using ΔE. Photodecomposition of MB is negligible in a blank sample without synthesized nanoparticles. The samples treated with sonosynthesis demonstrated the highest self-cleaning performance under sunlight. This was indicated by an ΔE of 45.9 for the discoloration of methylene blue, compared to the control sample (ΔE of 15.7), and the sample treated by the stirrer method with ΔE of 28.7. This is due to a reduction in particle size created by acoustic wave-controlled reaction, as well as the lower surface free energy of nanoparticles [40]. N–Ag/TiO_2_/ZnO nanocomposite particles were uniformly distributed on the sonotreated sample as confirmed by FE-SEM images and X-ray mapping. According to EDX analysis, the percentage of Zn, Ti, and Ag on Ag/Ti/Zn-stirred were lower than Ag/Ti/Zn1, indicated the ultrasound irradiation effect in the formation N–Ag/TiO_2_/ZnO composite. The ultrasonic wave assisted the reaction, resulting in well-controlled nanoparticles and cavitation which explains the smaller particle size of the N–Ag/TiO_2_/ZnO nanocomposite. The particle size distribution and shape of robust N–Ag/TiO_2_/ZnO photocatalyst prepared by in situ photo sonochemical method compared with conventional method indicate sonochemistry as a convenient method for achieving spherical cluster N–Ag/TiO_2_/ZnO particles on the surface of wool fabric. Among the various samples, the N–Ag/TiO_2_/ZnO hybrid composite demonstrated higher ΔE or superior self-cleaning performance relative to pristine photocatalysts (Table 3). According to the results, the optimized samples were significantly self-cleaned after seven days of daylight illumination, proving in situ photosynthesis N–Ag/TiO_2_/ZnO structure on wool surface for MB discoloration. The composite photocatalytic performance, illustrated schematically in Fig. 6, is likely due to its hybrid structure, enabling efficient charge separation. Upon exposure composite sample to photons with energies surpassing its band gap, electrons move to the conduction band, leaving holes in the valence band. The absorption of visible light excites both N-doped TiO_2_ and N-doped ZnO. Incorporating silver into the Ti–Zn hybrid sample enhanced sunlight utilization and reduced the recombination of photogenerated charge carriers due to its role as an electron trap and the formation of a Schottky junction barrier. The plasmonic properties of Ag boosted photocatalytic behavior by enhancing charge separation and extending light sensitivity into the visible range. Moreover, the incorporation of nitrogen doping increases active sites and extended visible light absorption by shifting absorption threshold to higher wavelengths. Nitrogen doping facilitated the transition of excited electrons to lower energies, expanding light absorption into the visible spectrum [29,30,32]. As a result, the high self-cleaning performance of the nanocomposite could be attributed to the synergetic impact of synthesized metal oxide semiconductors and noble metal in the following steps: first, formation of electron-hole pair and silver nanoparticles with surface plasmon resonance effect, second transmittance of the trapped electron to the conduction band of the TiO_2_ and ZnO, then transference photogenerated holes on synthesized photocatalysts, finally oxidation and photodegradation MB dye into CO_2_ and H_2_O [54,55].

Photocatalytic coatings on substrates other than textiles have also demonstrated significant self-cleaning activities. Hot et al. fabricated photocatalytic coated mortar samples with ZnO and TiO_2_ and evaluated their self-cleaning performance through color change measurements. The coated samples exhibited higher ΔE compared to the non-coated samples [56]. Incorporating TiO_2_ into a cementitious substrate showed high self-cleaning activity, with optimized additives to enhance moisture stability and mechanical properties [57].

Quantifying color changes in the CIELab space is commonly used in reports to study the degradation of a stain on a substrate. These color changes cannot be solely attributed to the photocatalytic self-cleaning properties but also to changes in the color of the substrate. Here, the control wool sample color changed due to the sunlight exposure.

### 4.3 UV protection ability

Untreated and treated samples were studied using 200–800 nm reflectance spectra to determine their UV-blocking ability, Fig. 5. The light reflection threshold in the composite was observed to peak at a wavelength higher than that of the other samples due to N doping and silver plasmonic effect [32,58]. Additionally, the optical reflection of N–Ag/TiO_2_/ZnO composite exhibited a significant decrease in the UV region compared to the other samples confirming the higher UV-blocking activity of the hybrid composite. UV blocking mechanism of semiconductor materials is correlated with their band gap energy and electronic properties. ZnO and TiO_2_ have band gaps in the UV region of the solar spectrum, corresponding to wavelengths of 365 and 380 nm, respectively. Under these wavelengths, electrons are excited by UV irradiation, which are absorbed by nanoparticles and provide UV protection for fabrics [59]. The reflectance of the non-treated sample in the UV region ranged from 5 to 20 % within 200–380 nm, while the reflectance of the N–Ag/TiO_2_/ZnO sonotreated sample remained constant at 4 %. This aligns with other findings on the UV absorption of photocatalyst-coated textiles, which protect the fabric surface from UV degradation and yellowing [1,17,19].

### 4.4 Inherent properties of the fabric

As shown in Table 4, the synthesis of ZnO and TiO_2_ nanoparticles on wool fabric increased tensile strength. Among the treated samples, the N–Ag/TiO_2_/ZnO composite showed the greatest enhancement of strength. In wool fibers, ultrasound irradiation pushes nanoparticles into keratin chains, which prevents wool fibers from being attacked and degraded by alkalis. The cross-linking effect of silver nanoparticles on proteins increases the tensile strength of modified fabric [19,60].

Fabricating nanocomposite on the sample surface increased the char residual percentage and flame retardant properties (Table 4). The yellowness index of the fabric was also decreased by nanoparticles coated on its surface. It was confirmed that ZnO, TiO_2_, and Ag had been synthesized on fabric samples based on a decreasing yellowness index and an increasing char residual.

Keratin disulfide bonds are decomposed by sulfhydryl anions formed in the alkaline region. The alkali solubility of samples modified with the N–Ag/TiO_2_/ZnO composite is lower than that of other treated samples, likely attributed to the potential cross-linking effect of the synthesized nanoparticles.

In acid hydrolysis, ZnO decomposes through topochemical degradation. However, adding TiO_2_ and Ag particles to ZnO leads to the formation of a self-supporting composite that exhibits superior acid resistance. Ultrasound-treated fabric exhibited lower levels of acid solubility than stirred one because the nanoparticles were distributed more evenly.

The water drop absorption rate of N–Ag/TiO_2_/ZnO sonotreated fabric increases rapidly compared with stirred, TiO_2,_ and ZnO coated samples, proving that the synergistic effect of metal-oxides and silver results in superior hydrophilicity. The hydrophilicity of fabric plays a critical role in the breakdown of organic dyes, then synthesizing nanocomposite may be an effective way of improving fabric hydrophilicity and thereby enabling better self-cleaning.

### 4.5 Antimicrobial and cytotoxicity

Cell viability, antibacterial activity, and antifungal activity of treated and untreated samples were studied and the results are presented in Table 4 highlighting the importance of controlling the size of the particle and the limitation of using different ranges of particle size distribution. The N–Ag/TiO_2_/ZnO sonotreated composite wool demonstrated improved antimicrobial and antifungal properties, achieving a 100 % reduction in two different bacteria, *E. coli,* and *S. aureus,* and a 99 % reduction in the fungus *C. albicans*. Furthermore, the nanoparticles exhibited low cytotoxicity, as indicated by a 70 % cell viability. Utilizing an ultrasound bath led to lower cell viability than stirred treated samples due to finer particles entering the cells and disturbing the natural metabolism. At the same time, finer-sized compounds introduce more antibacterial and antifungal activity than the usual ones.

Studies reported the antimicrobial activity of photocatalysts on various substrates as well. Carbon-doped TiO_2_-based photocatalytic paint was designed to control fungal growth under various light sources, resulting in 1.4 times greater fungus inactivation under UV light compared to regular paints [61]. The photocatalytic cement also showed antimicrobial activity with large inhibition zones (>22 mm). These zones are measured to determine how well a substance can stop bacteria from growing in the area around it [57].

In this study, the development of N–Ag/TiO_2_/ZnO-coated wool offers significant environmental benefits by degrading pollutants under natural sunlight, making it suitable for eco-friendly textile applications. Its enhanced antimicrobial properties make it a good candidate for healthcare uses. The inherent problematic properties of wool fabric were also modified. The presence of nanoparticles reduced the photo-yellowing of wool under UV light due to their UV-absorbing capability. Additionally, the alkali solubility of wool decreased with the incorporation of nanoparticles. The hydrophilicity of the naturally hydrophobic wool textile was enhanced, resulting in a decreased water absorption time. However, challenges remain in scaling up the lab-scale synthesis for industrial production while maintaining the same quality. Additionally, the long-term durability of the active wool, particularly resistance to repeated washing and environmental exposure, needs a thorough evaluation.

## 5 Conclusion

This study utilized ultrasonication and UV-irradiation, to synthesize composite photocatalysts and develop multifunctional fabric. N–Ag/TiO_2_/ZnO were introduced into the wool fabric surface through sonosynthesis and stirring methods, with optimal processing conditions determined using CCD. Among the treated samples, those subjected to sonosynthesis exhibited the highest self-cleaning performance under sunlight irradiation, as evidenced by an ΔE of 45.9 for the discoloration of methylene blue, compared to 15.7 for the control and 28.7 for the stirrer method. The enhanced self-cleaning activity was achieved by the photochemical degradation of methylene blue, attributed to the surface plasmon resonance of silver nanoparticles, nitrogen doping, and the hybrid photocatalyst with a narrower bandgap. Nanocomposites of N–Ag/TiO_2_/ZnO also improved the tensile strength of the textile by 20 % and reduced its susceptibility to acid and alkali solubility by promoting cross-linking between wool groups and nanoparticles. Sonotreated N–Ag/TiO_2_/ZnO samples demonstrated UV-blocking properties with reflectance remaining constant at 4 % from 200 to 380 nm, and enhanced hydrophilicity. Moreover, the antimicrobial and antifungal properties of the treated fabric were significantly enhanced, showing 100 % bacteria reduction against *E. coli* and *S. aureus*, and 99 % against *C. albicans* fungus. The 70 % cell viability revealed the low cytotoxicity behavior of the employed nanoparticles. This study underscores the importance of developing multifunctional textiles capable of addressing pollution, microbial threats, and UV radiation.

## CRediT authorship contribution statement

**Amir Behzadnia:** Writing – original draft, Visualization, Investigation, Data curation. **Majid Montazer:** Writing – review & editing, Visualization, Validation, Supervision, Resources, Project administration, Formal analysis, Conceptualization. **Mahnaz Mahmoudi Rad:** Validation, Supervision, Investigation, Data curation. **Madineh Rastgoo:** Writing – review & editing, Formal analysis.

## Declaration of competing interest

The authors declare that they have no known competing financial interests or personal relationships that could have appeared to influence the work reported in this paper.
